# Transcriptional response of Atlantic salmon families to *Piscirickettsia salmonis* infection highlights the relevance of the iron-deprivation defence system

**DOI:** 10.1186/s12864-015-1716-9

**Published:** 2015-07-04

**Authors:** Rodrigo Pulgar, Christian Hödar, Dante Travisany, Alejandro Zuñiga, Calixto Domínguez, Alejandro Maass, Mauricio González, Verónica Cambiazo

**Affiliations:** Laboratorio de Bioinformática y Expresión Génica, Instituto de Nutrición y Tecnología de los Alimentos (INTA), Universidad de Chile, El Líbano 5524, Santiago, Chile; Fondap Center for Genome Regulation, Av. Blanco Encalada 2085, Santiago, Chile; Center for Mathematical Modeling and Department of Mathematical Engineering, Av. Beauchef 851, Santiago, Chile

**Keywords:** Atlantic salmon families, *Piscirickettsia salmonis*, Infection susceptibility, Iron-deprivation, Gene expression

## Abstract

**Background:**

Piscirickettsiosis or Salmonid Rickettsial Septicaemia (SRS) is a bacterial disease that has a major economic impact on the Chilean salmon farming industry. Despite the fact that Piscirickettsia salmonis has been recognized as a major fish pathogen for over 20 years, the molecular strategies underlying the fish response to infection and the bacterial mechanisms of pathogenesis are poorly understood. We analysed and compared the head kidney transcriptional response of Atlantic salmon (Salmo salar) families with different levels of susceptibility to P. salmonis infection in order to reveal mechanisms that might confer infection resistance.

**Results:**

We ranked forty full-sibling Atlantic salmon families according to accumulated mortality after a challenge with *P. salmonis* and selected the families with the lowest and highest cumulative mortalities for microarray gene expression analysis. A comparison of the response to *P. salmonis* infection between low and high susceptibility groups identified biological processes presumably involved in natural resistance to the pathogen. In particular, expression changes of genes linked to cellular iron depletion, as well as low iron content and bacterial load in the head kidney of fish from low susceptibility families, suggest that iron-deprivation is an innate immunity defence mechanism against *P. salmonis*. To complement these results, we predicted a set of iron acquisition genes from the *P. salmonis* genome. Identification of putative Fur boxes and expression of the genes under iron-depleted conditions revealed that most of these genes form part of the Fur regulon of *P. salmonis.*

**Conclusions:**

This study revealed, for the first time, differences in the transcriptional response to *P. salmonis* infection among Atlantic salmon families with varied levels of susceptibility to the infection. These differences correlated with changes in the abundance of transcripts encoding proteins directly and indirectly involved in the immune response; changes that highlighted the role of nutritional immunity through iron deprivation in host defence mechanisms against *P. salmonis*. Additionally, we found that *P. salmonis* has several mechanisms for iron acquisition, suggesting that this bacterium can obtain iron from different sources, including ferric iron through capturing endogenous and exogenous siderophores and ferrous iron. Our results contribute to determining the underlying resistance mechanisms of Atlantic salmon to *P. salmonis* infection and to identifying future treatment strategies.

**Electronic supplementary material:**

The online version of this article (doi:10.1186/s12864-015-1716-9) contains supplementary material, which is available to authorized users.

## Background

The aquaculture industry has confronted severe economic losses due to infectious diseases in recent years [1, 2]. Therefore, control methods against infections and a thorough comprehension of resistance mechanisms are needed. Salmonid Rickettsial Septicaemia (SRS) is a bacterial disease that accounts for annual losses of over US$100 million in the Chilean salmon farming industry [3, 4]. *Piscirickettsia salmonis*, the causative agent of SRS, was first identified as a pathogenic agent in disease outbreaks among farmed Chilean Coho salmon (*Oncorhynchus kisutch*) in 1989, and since then, infectivity has been demonstrated in all cultured salmonid species, from the south of Chile to the Northern Hemisphere [5–7]. This pathogen has the ability to infect, replicate, and propagate in salmonid monocytes/macrophages [8, 9], and it produces a systemic infection characterized by the colonization of several organs, including the kidney, liver, spleen, intestine, brain, ovary, and gills [5, 10]. To date, the mechanisms employed by *P. salmonis* to infect, survive, and proliferate within host cells remain unknown.

Studies on the fish response to this pathogen have principally analysed changes in gene expression following bacterial infection [11, 12]. Rise et al. [11] reported altered expression levels of transcripts encoding proteins with roles in the antioxidant response and innate immunity in Atlantic salmon (*Salmo salar*) macrophages (24 h post-injection, hpi) and head kidney cells (14 days post-injection, dpi) with *P. salmonis* infection [11]. Likewise, Tacchi et al. [12] analysed the early response (48 hpi) to *P. salmonis* in the liver, head kidney cells, and muscle of Atlantic salmon and found activated inflammatory and oxidative defence response mechanisms. Thus, transcriptome analyses have provided evidence of cellular processes that may play a role in the host response to *P. salmonis* infection.

Recent studies have reported significant genetic variation for resistance to *P. salmonis* infection in Atlantic salmon families [13, 14], and heritability estimates range from 0.11 to 0.41 (medium/high magnitude), indicating that this trait might be genetically improved. Therefore it is of particular interest to examine the transcriptomic response of Atlantic salmon to *P. salmonis* infection considering genetic background. This approach will provide insight on the biological mechanisms that lead to genetic differences in resistance. In other economically important fish species, variances in gene expression between resistant and susceptible genetic groups have identified genes and pathways underlying differences in infection resistance [15–17].

In the present study, we analysed the transcriptional response to *P. salmonis* in groups of Atlantic salmon families with low or high susceptibility to infection. Our study aims to contribute to the current understanding of resistance mechanisms to *P. salmonis* infection.

## Results and discussion

### Differential susceptibility of Atlantic salmon families to *P. salmonis* infection

**Fig. 1:**
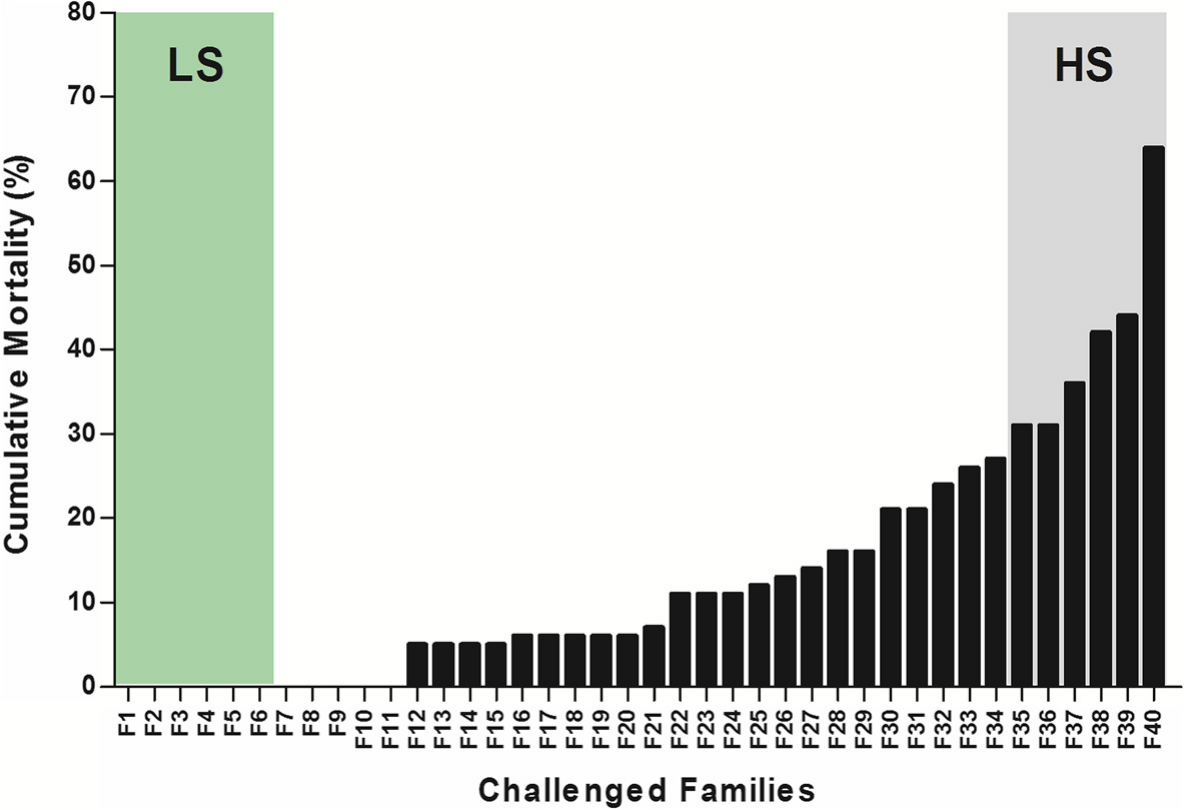
Cumulative mortality following *P. salmonis* infection. Forty full-sibling Atlantic salmon families were challenged for 40 days with an intra-peritoneal dose of *P. salmonis* (isolate PS889). Families were enumerated according to their cumulative percentages of mortality. The families with the highest mortality levels (31.3 – 64.3 %, grey column) were named families of high susceptibility (HS), while the families with the lowest mortality levels (0 %, green column) were named families of low susceptibility (LS)

In this study, groups of approximately 20 fish from forty full-sibling Atlantic salmon families were used in controlled experimental challenges. Tagged fish were infected by intra-peritoneal injection (IP) with *P. salmonis* (1 × 10^4^ PFU/ml) and randomly distributed among ten tanks in order to reduce the possibility of bias during fish culturing and handling. Even though an IP challenge is not a natural form of infection, it is an effective method for presenting a naïve animal with a known and controlled amount of bacteria [11, 12]. Cumulative fish mortality was used as a measure of susceptibility to *P. salmonis* as survival reflects the cumulative effects of all host-pathogen interactions during infection and is therefore the best criteria to determine the level of susceptibility [18]. The cumulative mortalities of fish families injected with *P. salmonis* ranged from 0 to 64.3 % at 40 dpi (Fig. 1), with most families presenting cumulative mortalities between 5 and 16 % (*n* = 28). In families with some degree of mortality, fish showed several clinical signs characteristic of infection: lethargy, anorexia, darkening of the skin, respiratory distress, and/or surface swimming [5]. To confirm the presence of *P. salmonis* in the challenged fish, the 16S rRNA gene of *P. salmonis* was quantified using real-time PCR (qPCR) in at least three fish from each family. Results indicated that the bacterium was present in the head kidney and spleen of all challenged fish, whereas unchallenged control fish presented negative results (data not shown). Based on cumulative mortality data, two groups of families were defined (Fig. 1). The low susceptibility (LS) group was comprised of families with cumulative mortalities of 0 %, and the high susceptibility (HS) group included families with a cumulative mortality greater than 30 %. In both groups, mortality caused by *P. salmonis* began 15 to 16 dpi (data not shown), but variances in cumulative mortality were evidenced by the end of the first challenge. The results of the first challenge indicated a differential distribution of cumulative mortality among infected families, suggesting that the susceptibility of fish families to *P. salmonis* infection is a result of differences in the genetic backgrounds.

The influence of genetic factors on fish susceptibility to *P. salmonis* [13, 14] and other bacterial infections has been previously reported in other fish species [15, 17, 19, 20]. For example, Camp et al. [20] challenged fifteen full-sibling families of juvenile channel catfish (*Ictalurus punctatus*) with the bacterium *Edwardsiella ictaluri*, the causative agent of enteric septicemia of catfish. Cumulative mortalities among families ranged from 5 to 35 %. Using the most resistant and sensitive families, they also detected differences between families in macrophage aggregations and in the amount of lymphocytes (B and T) in peripheral blood. In a different work, the expression of transcripts encoding for complement component 3 (C3) and lysozyme C II were induced to a greater extent in a resistant rainbow trout (*Oncorhynchus mykiss*) line compared to a susceptible line in response to *Flavobacterium psychrophilum* infection [15]. Moreover, changes in the expression of transcripts encoding several toll-like receptors and innate immune components were observed in genetic groups of catfish (*Ictalurus punctatus*) and Japanese flounder (*Paralichthys olivaceus*) with different susceptibilities to *Edwardsiella spp* infection [16, 17]. Thus, the results of these and other studies indicate an association between infection susceptibility levels in fish and changes in the relative abundances of transcripts involved in immune and stress responses, among other processes.

### Gene expression analysis of LS and HS families

For gene expression analysis, a second group of naïve fish belonging to the six most resistant and six most susceptible families (LS and HS, respectively) was IP injected with *P. salmonis*. Fish from each family were divided into control (non-infected fish, *n* = 10) and treated (infected fish, *n* = 10) groups, and head kidney samples from each fish were collected at 14 dpi, before the onset of mortality, and analysed using microarrays. No differences in fish weight between LS (487.5 ± 104.5 g) and HS (485.5 ± 122.6 g) or between control and infected groups were observed. Head kidney samples were also examined using qPCR assays to confirm the presence of *P. salmonis* in all IP-injected fish and its absence in control fish.

Three LS and three HS families were selected for microarray assays. For each assay, head kidney RNA from groups of five non-infected and five infected individuals were pooled, reverse transcribed, and hybridized to microarrays. In order to produce a general description of the transcriptional response to *P. salmonis* in fish families with different susceptibilities to the infection, each of the three LS families and each of the three HS families were regarded as biological replicates of the high and low susceptibility groups, respectively (experimental design in Additional file 1).

**Fig. 2:**
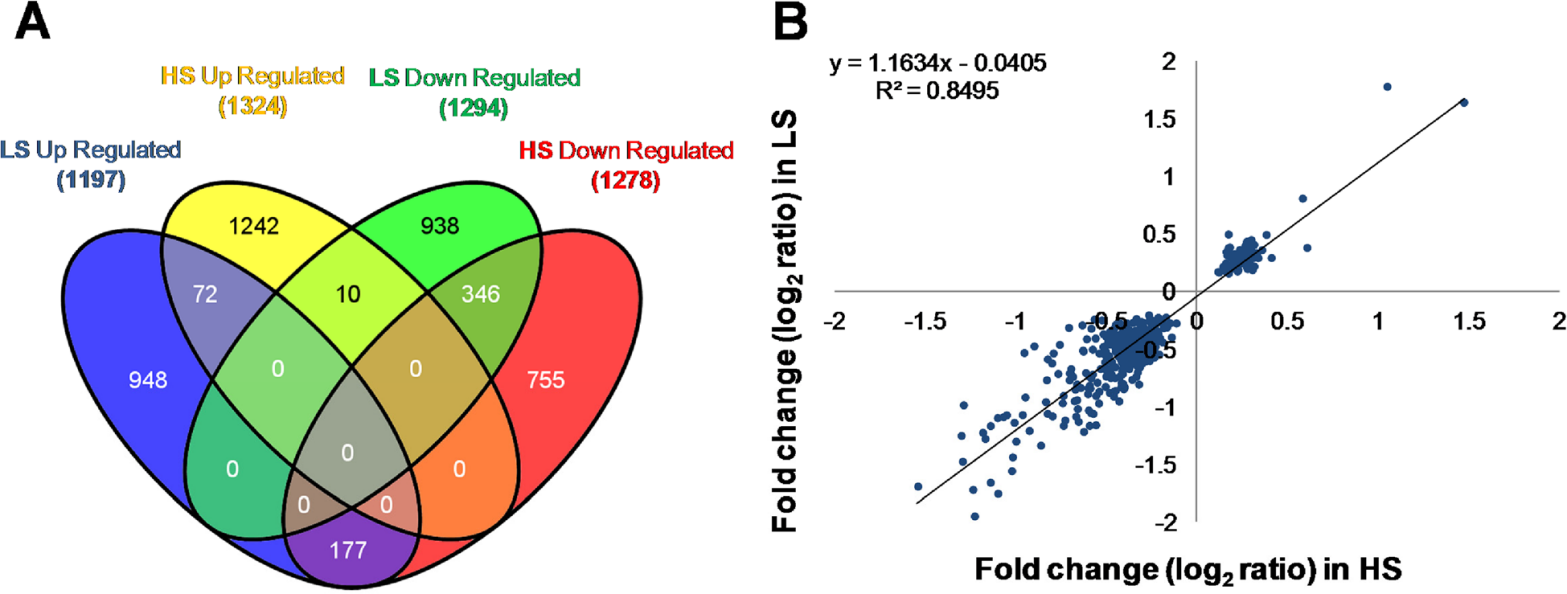
Global transcriptome response of fish head kidney to *P. salmonis* infection. **a** Venn diagram of differentially expressed probes between infected and control fish from LS and HS families. **b** Representation of Fold Change (log_2_) of all common probes significantly modulated by *P. salmonis* infection in LS (*X axis*) and HS (*Y axis*) families. Parameters of the linear regression are indicated

Results indicated that 2491 and 2602 probes were differentially expressed (false discovery rate (FDR) adjusted, *p* < 0.05) between infected and non-infected fish of the LS and HS groups, respectively (Fig. 2a). Fold change (FC) values of differentially expressed probes were well correlated (Pearson correlation = 0.92) between the two groups of families (Fig. 2b), indicating that the response to *P. salmonis* infection was similar in terms of these indicators. A complete list of probes differentially expressed following bacterial infection is shown in Additional file 2. Some of these probes contained different regions of the same genes or paralogs. A total of 1430 probe sets (735 up-regulated and 695 down-regulated) were differentially expressed between infected and non-infected fish of the LS group, of which 1288 probe sets corresponded to genes with predicted functions. For the HS group, statistically significant gene expression differences between infected and non-infected fish were observed for a total of 1300 probe sets (625 up-regulated and 675 down-regulated), of which 1154 corresponded to genes with predicted functions.

**Fig. 3:**
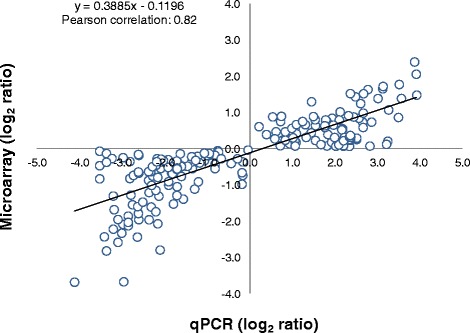
qPCR validation of microarray results. Mean log_2_ ratios (infected/control) of gene expression (*N* = 33, Additional file 3) calculated from microarrays were plotted against the mean log_2_ ratios derived from qPCR assays. Each circle represents the mean of five technical replicate (*N* = 990 assays). Correlation between microarrays and qPCR was calculated by Pearson product moment correlation and a *p* < 0.01 was considered statistically significant

To validate results from the microarrays, the relative transcript abundance of a random set of 40 genes was examined by qPCR. A microarray result was considered validated when the RNA expression profile of a gene is statistically differential in response to infection and followed the same trend when tested by both microarray and qPCR in the six families. Among the 40 genes that were subjected to validation, 33 (83 %) displayed the same trend observed in the microarray analysis of the six families (Additional file 3). Overall, a strong positive correlation of 0.82 (Pearson correlation) was determined between microarray and qPCR analyses for the combined data set (*p* < 0.00001) (Fig. 3). Thus, although qPCR showed a broader dynamic range than microarrays, these two platforms correlated well with each other.

### Common transcriptional response of LS and HS families to *P. salmonis* infection

We determined the number of common (shared) probe sets that were significantly up- (*n* = 54) or down-regulated (*n* = 155) in the two groups of families following *P. salmonis* infection; a list of representative shared genes is shown in Table 1 (see the complete list of probes in Additional file 4). Of the genes that increased their expression in response to infection, we detected functional categories that were associated with the antibacterial response, such as the immune response, energy metabolism, and cytoskeleton rearrangement, among others [21]. In particular, among transcripts encoding proteins with predicted roles in the innate immune response we found lysozyme C II (*lyz*), which has a hydrolytic activity against Gram-positive and Gram-negative bacteria in tissues and body fluids [22, 23]. Its increased transcript abundance and enzyme activity has been widely described in fish infected with different pathogens [16, 24, 25], indicating a conserved and relevant function in antimicrobial defence. Additionally, among the transcripts with functions involved in the adaptive immune response, one was found that encoded for a component of the major histocompatibility complex (MHC) class I (*hla-UBA*). Moreover, the expression of some components linked to the organization and regulation of the actin cytoskeleton, such as cytoplasmic actin, thymosin, tropomyosin, and myosin light chains, were also up-regulated. In this regard, dynamic rearrangements and the organization of the actin cytoskeleton are critical for lymphocyte migration, as well as for the formation and stabilization of the immunological synapse at the interface between antigen-presenting cells and T cells [26].

**Table 1:** Representative probe sets differentially expressed between infected and non-infected fish

GeneBank ID^a^	Fold change^b^		Tentative annotation^c^	Adj. P value^d^	
Up-regulated in LS and HS families	LS	HS		LS	HS
Immune response					
CB511680	1.468	1.642	Lysozyme C II	0.000	0.000
CA050178	0.171	0.497	MHC class I (hla-UBA)	0.022	0.008
CA063704	0.114	0.171	CD97 antigen	0.045	0.030
CB508464	0.329	0.311	Canopy 4 (cnpy4)	0.028	0.032
CA044420	0.408	0.293	Aminopeptidase N	0.024	0.044
CB493358	0.288	0.373	Microsomal glutathione S-transferase 2	0.048	0.011
Energetic metabolism					
CB500248	0.266	0.378	ATP synthase subunit e, mitochondrial	0.023	0.007
CA768741	0.273	0.301	ATP synthase subunit epsilon, mitochondrial	0.008	0.013
CA045510	0.252	0.252	NADH dehydrogenase [ubiquinone] 1 alpha subcomplex subunit 1	0.009	0.013
CK991263	0.169	0.219	NADH dehydrogenase [ubiquinone] 1 beta subcomplex subunit 8	0.039	0.007
CB493482	0.251	0.276	Glucagon-1	0.014	0.005
CB492183	0.289	0.243	Succinyl-CoA ligase beta-chain	0.007	0.040
CB497724	0.225	0.280	Mitochondrial import receptor subunit TOM7	0.041	0.046
Organization and regulation of actin cytoskeleton					
CA770217	0.160	0.345	Actin, cytoplasmic 1	0.019	0.005
CB508611	0.264	0.293	Thymosin beta-4	0.015	0.018
EG804880	0.231	0.294	Tropomyosin alpha-3 chain	0.037	0.012
EG783905	0.605	0.381	Myosin light chain 3	0.026	0.009
BU965651	0.326	0.337	Myosin light chain 4	0.010	0.012
DW564371	0.174	0.193	Ras GTPase-activating protein nGAP (ngap)	0.044	0.032
CB508887	0.248	0.306	Ral guanine nucleotide dissociation stimulator	0.021	0.021
Cell metabolism and regulation					
CB507177	0.237	0.356	Cell division protein kinase 10	0.018	0.015
DW577024	0.198	0.304	Anaphase-promoting complex subunit 13	0.048	0.009
CB500559	0.227	0.251	Casein kinase II subunit alpha	0.009	0.017
CB492123	0.276	0.196	Trafficking protein particle complex subunit 5	0.025	0.040
CA042337	0.156	0.174	Charged multivesicular body protein 3	0.034	0.041
CB486725	0.202	0.326	Vacuolar ATP synthase subunit S1	0.029	0.009
CB490914	0.252	0.309	Fumarylacetoacetase	0.040	0.008
CB492396	0.199	0.277	Cytosolic 5-nucleotidase III	0.046	0.024
Stress response					
CA045475	0.313	0.407	Pro-opiomelanocortin	0.026	0.004
CA047150	0.334	0.292	Pro-opiomelanocortin A2s	0.013	0.009
CA054693	0.157	0.246	Alcohol dehydrogenase class-3	0.048	0.020
EG824838	0.184	0.297	Microsomal glutathione S-transferase 3	0.033	0.022
CB498572	0.245	0.246	Gamma-glutamyl hydrolase	0.017	0.027
Gene expression					
EG812701	0.579	0.809	Histone deacetylase complex subunit SAP30L	0.000	0.020
CA045554	0.310	0.332	Neurogenic differentiation factor 1	0.026	0.012
CB493607	0.313	0.219	Small nuclear ribonucleoprotein-associated protein B	0.002	0.013
CA058810	0.231	0.243	Eukaryotic translation initiation factor 1A	0.014	0.011
CA060458	0.297	0.449	Eukaryotic translation initiation factor 5A-1	0.023	0.012
Down-regulated in LS and HS families	LS	HS		LS	HS
Synthesis of proteins					
CA045933	−0.703	−0.707	40S ribosomal protein S27	0.000	0.002
CA051651	−0.562	−0.403	40S ribosomal protein S5	0.008	0.026
CB497637	−0.610	−0.409	40S ribosomal protein S19	0.017	0.025
CA037570	−0.590	−0.840	60S ribosomal protein L27	0.002	0.000
CA768633	−0.585	−0.318	60S ribosomal protein L38	0.005	0.031
CB501170	−0.629	−0.517	60S ribosomal protein L36	0.002	0.001
Glycolysis, Oxidative Phosphorylation and mitochondrial transport					
CA768062	−0.318	−0.654	Glyceraldehyde-3-phosphate dehydrogenase	0.005	0.017
CA052837	−0.424	−0.497	Phosphoglycerate mutase 1	0.006	0.025
CN442520	−1.051	−1.070	Cytochrome oxidase subunit 2	0.001	0.000
CB493612	−0.461	−0.909	Cytochrome oxidase subunit 3	0.014	0.001
CN442526	−0.311	−0.420	Cytochrome b	0.029	0.009
CA063030	−0.708	−0.311	NADH dehydrogenase [ubiquinone] 1 alpha subcomplex subunit 6	0.038	0.048
CN442551	−0.826	−1.058	NADH-ubiquinone oxidoreductase chain 1	0.001	0.008
CN442556	−0.416	−0.476	NADH-ubiquinone oxidoreductase chain 2	0.000	0.043
BU965678	−0.926	−1.207	NADH-ubiquinone oxidoreductase chain 3	0.000	0.008
CN442494	−0.320	−0.528	NADH-ubiquinone oxidoreductase chain 4	0.043	0.019
CA042906	−0.454	−0.746	ADP/ATP translocase 2	0.027	0.014
CA058445	−0.187	−0.313	ADP/ATP translocase 2	0.034	0.009
CB502545	−0.399	−0.595	Mitochondrial import inner membrane translocase subunit Tim23	0.043	0.000
CB498852	−0.402	−0.828	Heat shock cognate 70 kDa protein	0.031	0.000
Plasma transport of oxygen and selenium					
CB492263	−1.300	−1.249	Hemoglobin subunit alpha-4	0.013	0.009
BU965636	−1.140	−1.657	Hemoglobin subunit beta	0.044	0.003
CA049300	−1.540	−1.691	Hemoglobin subunit beta-1	0.016	0.010
CB498665	−1.140	−1.164	Hemoglobin subunit beta-4	0.035	0.042
CA044104	−1.227	−1.947	Selenoprotein Pa	0.000	0.000
CB510462	−0.463	−0.867	Selenoprotein Pb	0.015	0.000
Extracellular matrix organization and collagen biosynthesis					
CA047568	−0.216	−0.223	Collagen alpha-1(I) a chain	0.024	0.046
CB488336	−0.427	−0.272	Collagen alpha-1(I) a chain	0.010	0.026
CA061635	−0.568	−0.239	Collagen alpha-1(I) b chain	0.001	0.046
CB493159	−0.296	−0.309	Collagen alpha-2(I) chain	0.005	0.021
CB492428	−0.246	−0.478	Secreted protein acidic and rich in cysteine (SPARC)	0.042	0.030
Homeostasis of metals (iron and copper)					
CB509708	−0.353	−0.443	Ferritin, heavy subunit	0.002	0.005
CB510731	−0.376	−0.634	Ferritin, middle subunit	0.001	0.006
CA039497	−0.337	−0.736	Hemopexin	0.005	0.041
CB508872	−0.767	−0.712	Metallothionein A	0.009	0.029
CB507722	−0.245	−0.328	Metallothionein B	0.048	0.009

^a^GenBank accession numbers of probes

^b^Fold change between infected and non-infected fish represented as log_2_ of expression ratio

^c^Tentative annotation of the best match according to blastx or blastn against nr GenBank database

^d^An adjusted *p* < 0.05 indicated that gene expression level was significantly altered following bacterial infection. Complete list of differentially expressed probes is available in Additional file 2

On the other hand, genes that had a significantly lower expression in response to infection were associated with different cellular processes and provided some insights on how this pathogen modulates the host response. Thus, transcripts that showed decreased relative abundance following bacterial infection were mainly involved in the processes of protein synthesis (ribosomal proteins), transport of oxygen and selenium, and homeostasis of metals. Twenty-four probe sets encoding 40S ribosomal subunit proteins and 38 encoding 60S ribosomal subunit proteins were down-regulated in response to infection, suggesting that the transcriptional repression of translation machinery might be an antibacterial response or part of a general reduction in host metabolic activity. It has been suggested that the shut-down of translation machinery is a bacterial and viral strategy to control the translation of pathogenic proteins [27–30] and to suppress innate host defences by inhibiting the capacity of infected cells to synthesize immune system proteins [31, 32]. This strategy may be used by *P. salmonis* to control the host response in order to survive and replicate inside infected cells [8]. Since large numbers of probe sets represent the same protein or processes, these results suggest a coordinated gene expression response to *P. salmonis* infection.

The relative abundance of transcripts encoding for hemoglobin subunits and selenoprotein P was also significantly decreased in response to infection, suggesting that *P. salmonis* might impact the plasma transport of oxygen/iron and selenium. The down-regulation of hemoglobin subunits may be part of a host defence mechanism to limit the availability of hemic-iron, an important source of iron for intracellular bacteria [33–35]. Moreover, decreased relative abundance of transcripts encoding for proteins involved in intracellular non-hemic iron binding (ferritin middle and heavy subunits) and in hemic binding (hemopexin) suggest that *P. salmonis* infection induces changes in iron metabolism in Atlantic salmon. These changes may affect the expression of genes directly involved in the synthesis of hemoglobin, as has been reported for other pathogens [36]. Finally, Selenoprotein P, an extracellular protein that transports most plasma selenium [37], was consistently down-regulated following infection. Selenoprotein P has been associated with oxidative [38] and immune defence [39] mechanisms, and it has been proposed as a viable candidate molecular marker for responses to *P. salmonis* [11] and anaemia virus (ISAv) [40] infections. Thus, the down-regulation of Selenoprotein P transcripts seems to be a conserved response to different types of pathogens infecting Atlantic salmon.

Since knowing the processes in which differentially expressed genes are involved helps to understand the host-pathogen interaction, a functional analysis was used to identify biological processes (Gene Ontology and Reactome) and metabolic pathways (KEGG) in all probe sets, including those that were mutually or distinctly up- or down-regulated in the family groups (Table 2). Functional annotation of genes with increased expression highlighted the central place of immune processes, such as toll-like receptor signalling, bacterial infection (*Salmonella)*, and phagosome/lysosome pathways, which are crucial for innate immune responses involved in the recognition, phagocytosis, and degradation of pathogens [41, 42]. Genes with decreased expression in both LS and HS families were mainly annotated to functional categories associated with protein complex assembly and translation. This result was consistent with that obtained with the functional analysis of shared down-regulated genes, and it strengthens the idea that *P. salmonis* hijacks the translation machinery of the host cell. In addition to this, hemopoiesis was another common functional term among down-regulated probes, and this might reflect the fish response to anaemia induced by *P. salmonis* infection (Table 2).

**Table 2:** Functional annotation of common probes significantly up- or down-regulated in LS and HS groups of families

Up-regulated in LS and HS families				
^a^Ontology ID	^b^Ontology Source	^c^Ontology Term	^d^Ontology Levels	^e^Number of genes
GO:0000226	GO_BiologicalProcess	microtubule cytoskeleton organization	[3, 6]	8
GO:0019318	GO_BiologicalProcess	hexose metabolic process	[5, 6]	8
GO:0071310	GO_BiologicalProcess	cellular response to organic substance	[4]	11
GO:0051603	GO_BiologicalProcess	proteolysis involved in cellular protein catabolic process	[5, 6]	17
KEGG:00190	KEGG	oxidative phosphorylation	[−1]	22
KEGG:04142	KEGG	lysosome	[−1]	10
KEGG:04145	KEGG	phagosome	[−1]	11
KEGG:05132	KEGG	Salmonella infection	[−1]	7
KEGG:04620	KEGG	Toll-like receptor signaling pathway	[−1]	9
REACTOME:2756221	REACTOME	metabolism of lipids and lipoproteins	[−1]	26
Down-regulated in LS and HS families				
^a^Ontology ID	^b^Ontology Source	^c^Ontology Term	^d^Ontology Levels	^e^Number of genes
GO:0006325	GO_BiologicalProcess	chromatin organization	[6]	7
GO:0006461	GO_BiologicalProcess	protein complex assembly	[4, 5]	8
GO:0030097	GO_BiologicalProcess	hemopoiesis	[4–6]	10
GO:0042981	GO_BiologicalProcess	regulation of apoptotic process	[5, 6]	11
KEGG:00480	KEGG	Glutathione metabolism	[−1]	8
REACTOME:2756367	REACTOME	Phospholipid metabolism	[−1]	10
REACTOME:2756199	REACTOME	Glycolysis	[−1]	5
REACTOME:2756244	REACTOME	mRNA Splicing - Major Pathway	[−1]	13
REACTOME:2756574	REACTOME	Eukaryotic Translation Elongation	[−1]	56

^a^Ontology accession numbers (ID)

^b^Ontology Source, Biological Process (GO), KEGG and Reactome

^c^Ontology Term and

^d^Ontology Level associated

^e^Number of genes found in a given ontology category within the analysed list of target probes

### Gene expression differences in LS and HS families following the *P. salmonis* infection

We hierarchically clustered the complete list of probes differentially expressed between infected and non-infected fish from the LS and HS families (Additional file 2 and Additional file 4), and a correlation analysis was applied to measure the degree of association among the gene expression patterns of the six families. A Pearson correlation (Additional file 5: Figure S1A) and Euclidian distance (Additional file 5: Figure S1B) were used as metrics, and an average linkage clustering described the data. Both clustering analyses clearly separated the three LS families from the three HS families in two distinct branches and showed a close proximity among families with similar levels of susceptibility to infection. These results suggest that salmon families with different levels of susceptibility to the infection differentially modulate transcript abundance in response to the pathogen.

To further examine the varied responses to *P. salmonis* infection between LS and HS families, and to identify potential mechanisms of natural resistance, we analysed the biological processes and metabolic pathways in two groups of probe sets differentially expressed between infected and non-infected fish (Table 3). The first group included 1138 probe sets that were significantly modulated (up- or down-regulated) in the LS but not the HS group. The second group included 127 probe sets that were up-regulated in LS families but down-regulated in HS families. Analysis of these underlined the central place occupied by both innate and adaptive immune systems, as represented by transcripts encoding for complement proteins (C3, C4, factor B), Myeloperoxidase (MPO), CXC and CC chemokines and receptors, interleukin 18b, and immunoglobulins (Additional file 6). Interestingly, whereas the alpha polypeptide of MHC class I was up-regulated in both LS and HS families, β2-microglobulin, another polypeptide of MHC class I, was consistently down-regulated only in HS families.

**Table 3:** Functional annotation of genes differentially expressed between LS and HS in response to *P. salmonis* infection

Up-regulated in LS families but not modulated in HS Families				
^a^Ontology ID	^b^Ontology Source	^c^Ontology Term	^d^Ontology Levels	^e^Number of genes
GO:0016052	GO_BiologicalProcess	carbohydrate catabolic process	[3, 4]	9
GO:0030036	GO_BiologicalProcess	actin cytoskeleton organization	[3, 6]	12
GO:0016570	GO_BiologicalProcess	histone modification	[6, 9]	4
REACTOME:2756220	REACTOME	Fatty acid, triacylglycerol, and ketone body metabolism	[−1]	11
REACTOME:2756441	REACTOME	Innate Immune System	[−1]	15
Down-regulated in LS families but not modulated in HS Families				
^a^Ontology ID	^b^Ontology Source	^c^Ontology Term	^d^Ontology Levels	^e^Number of genes
GO:0006413	GO_BiologicalProcess	translational initiation	[2, 5, 6]	6
GO:0006935	GO_BiologicalProcess	chemotaxis	[3, 4]	6
GO:0006875	GO_BiologicalProcess	cellular metal ion homeostasis	[6, 8]	6
KEGG:04150	KEGG	mTOR signaling pathway	[−1]	6
KEGG:04070	KEGG	Phosphatidylinositol signaling system	[−1]	6
KEGG:04650	KEGG	Natural killer cell mediated cytotoxicity	[−1]	6
REACTOME:2756528	REACTOME	Platelet activation, signaling and aggregation	[−1]	17
Up-regulated in LS families and Down-regulated in HS Families				
^a^Ontology ID	^b^Ontology Source	^c^Ontology Term	^d^Ontology Levels	^e^Number of genes
GO:0044262	GO_BiologicalProcess	Cellular carbohydrate metabolic process	[3, 4]	16
GO:0007010	GO_BiologicalProcess	cytoskeleton organization	[5]	10
GO:0006457	GO_BiologicalProcess	protein folding	[5]	12
GO:0015992	GO_BiologicalProcess	proton transport (ATP synthesis)	[4–7]	8
GO:0005506	GO_MolecularFunction	iron ion binding	[6]	12
KEGG:00980	KEGG	Metabolism of xenobiotics by cytochrome P450	[−1]	4
REACTOME:2756314	REACTOME	Adaptive Immune System	[−1]	21
REACTOME:2757180	REACTOME	Class I MHC mediated antigen processing & presentation	[−1]	12
REACTOME:2757144	REACTOME	Endosomal Sorting Complex Required For Transport (ESCRT)	[−1]	6
REACTOME:2756315	REACTOME	Immune System	[−1]	34
REACTOME:2756204	REACTOME	Metabolism of amino acids and derivatives	[−1]	15
REACTOME:2756598	REACTOME	Post-translational protein modification	[−1]	8
REACTOME:2756873	REACTOME	Regulation of Apoptosis	[−1]	9

^a^Ontology accession numbers (ID)

^b^Ontology Source, Biological Process (GO), KEGG and Reactome

^c^Ontology Term and

^d^Ontology Level associated

^e^Number of genes found in a given ontology category within the analysed list of target probes

It is worth mentioning that the characterization of host transcriptional changes at a late stage of *P. salmonis* infection (14 dpi) included genes directly involved in fighting infection, as well as genes involved in general physiological processes. Among these were genes with predicted functions in histone modification, protein folding, and carbohydrate and fatty acid metabolism (Table 3). This observation suggests that potential infection biomarkers could be involved in more general cellular processes and may not be limited to genes directly involved in the immune response.

### Differential activation of the iron-depletion system in LS and HS families after *P. salmonis* infection

The functional classification of genes differentially expressed between infected and non-infected fish revealed that part of the core response to *P. salmonis* infection included the down-regulation of several probes representing transcripts that encode for heme-proteins (hemoglobin and cytochrome), heme-binding proteins (hemopexin), and non-hemic iron binding proteins (ferritin, middle and heavy subunits). Moreover, transferrin, the iron-binding glycoprotein that transports iron in the plasma [43], was up-regulated in LS but down-regulated in HS families. However, hepcidin, the principal regulator of iron efflux in vertebrates, which controls access of iron into circulation [44, 45], was significantly up-regulated in HS families but not in LS families (Additional file 2). These results suggest that the regulation of iron homeostasis could be crucial for the natural resistance of Atlantic salmon to *P. salmonis* infection.

**Fig. 4:**
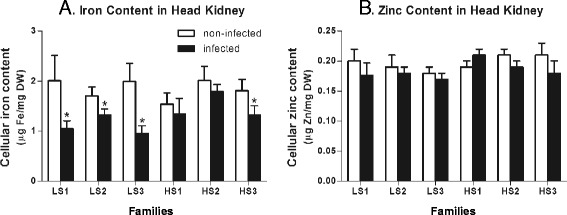
Cellular iron and zinc content in infected and non-infected fish from LS and HS families. **a** Cellular content of iron (μg) in dry weight (DW) head kidneys (mg) from non-infected (*white bars*) and infected tissues (*black bars*). **b** Cellular content of zinc (μg) in dry weight (DW) head kidneys (mg) from non-infected (*white bars*) and infected tissues (*black bars*). In all case, bars represent the mean of five biological replicate determinations (± SEM); *, *p* < 0.05 (Student’s *t* test)

Based on the central role that iron plays in both pathogen virulence and host anti-microbial resistance [46], we further examined whether differences in iron content and metabolism could be detected between LS and HS families in response to *P. salmonis* infection. To do this, the total iron content in non-infected and infected fish head kidneys was measured. Head kidney iron content ranged between 1.5 and 2.0 μg Fe/mg dry weights in non-infected fish from all families regardless of susceptibility (Fig. 4). However, at 14 dpi with *P. salmonis,* a significantly lower level of iron content was detected in infected fish from all LS families and in only one of the HS families (HS3) (Fig. 4a). Since there is evidence for a metabolic interaction between trace metals such as Fe and Zn [47, 48], Zn content was also assessed in infected fish families. The Zn content was similar in control fish of the LS and HS families, and levels were unaffected by infection (Fig. 4b). In view of the importance that iron availability has for bacterial proliferation [34, 49, 50], bacterial load was measured in infected tissues (Table 4). The results showed that the bacterial load was significantly lower in LS compared to HS families, indicating a positive correlation between the bacterial load and the iron content in kidneys after infection. This is a relevant aspect because it has been demonstrated that iron depletion limits intracellular bacterial growth in murine macrophage models [50].

**Table 4:** Bacterial load in infected fish

Family	Mean relative abundance	SEM
LS1	0.7	0.18
LS2	1.3	0.10
LS3	0.6	0.15
HS1	19.3	0.32
HS2	5.2	0.54
HS3	8.7	1.94

The bacterial load (in arbitrary units) was measured by qPCR in infected head kidneys from fish from LS and HS families. Results were normalized relative to the abundance of elongation factor 1 alpha (EF1A) transcript

As iron content between LS and HS families was similar without infection, the correlation between decreased metal content, reduced bacterial load, and pathogen resistance might be explained by an ability of LS families to reduce iron content in the head kidney in response to infection, and not by the iron status preceding infection. This suggests that decreased cellular iron content is a physiological response to infection.

**Fig. 5:**
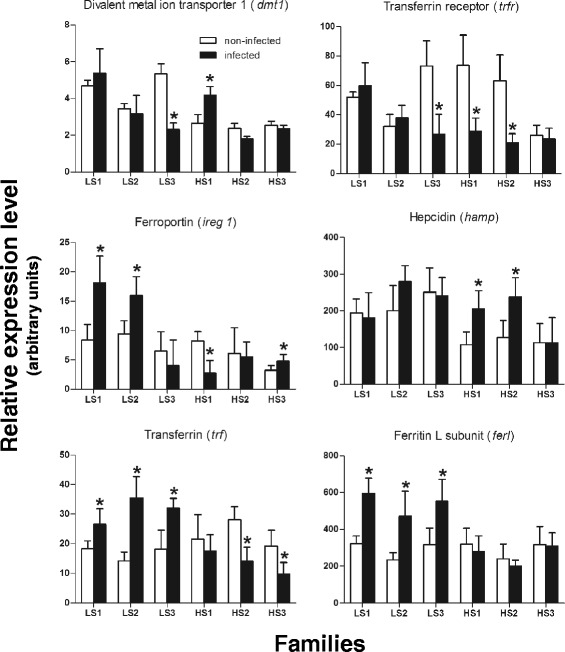
Expression analysis of iron metabolism genes in head kidneys from infected and non-infected fish. Relative changes in the expression of genes were determined using qPCR in non-infected (white bars) and infected fish (black bars) from LS and HS families. For each gene the relative abundance of mRNA was normalized towards the elongation factor 1 alpha (EF1A) mRNA. Bars represent the mean of five replicate determinations (± SD); *, *p* < 0.05 (Student’s *t* test)

To begin to understand the molecular mechanisms by which fish are able to reduce iron content in response to the infection, we first measured the relative abundance of transcripts with potential functions in iron transport and metabolism in both infected and non-infected fish kidneys from every LS and HS family. The data revealed that following infection, the LS3 family decreased the abundance of transcripts encoding iron uptake transporters (*dmt1*, divalent metal transporter 1; and *trfr*, transferrin receptor), whereas families LS1 and LS2 increased the abundance of transcripts encoding for the efflux transporter (*ireg1*, ferroportin) (Fig. 5). This might account for the reduced cellular iron measured after infection, as has been reported for *Salmonella* infection in murine macrophages [51].

Although HS families were also able to modify the abundance of transcripts encoding iron transporters after infection, in some cases, modulation was opposite to that observed in LS families. For example, the HS1 family (that most susceptible to infection, with 64.3 % cumulative mortality) showed a significant increase in the abundance of *dmt1* transcripts and a decrease in the abundance of transcripts encoding for Ireg1. The HS3 family, the only HS family that showed a significant decrease in iron content, displayed increased Ireg1 while no changes were detected for uptake transporters. On the other hand, the HS2 family had a decreased abundance of uptake transporters without alterations in the abundance of Ireg1. Since the HS2 family was not able to reduce cellular iron content in response to infection, this suggests that Ireg1activity may be pivotal to reduce iron availability in infected fish. Interestingly, the transcript encoding for the peptide hepcidin, which causes ferroportin internalization and degradation [44], was up-regulated only in those families that did not reduce iron abundance following infection, thus supporting the idea that the regulation of iron efflux is fundamental for reducing the intracellular content of this metal.

Finally, the effect of *P. salmonis* infection on the abundance of transferrin (*trf*) and ferritin light chain (*ferl*) transcripts was evaluated. These encode for ubiquitous proteins that bind and store extracellular and intracellular iron, respectively [52]. Results showed a significant increase in the abundance of *trf* and *ferl* in all LS families and a reduction of *trf* in two HS families, suggesting that infected fish were able to trigger strategies to limit the access of *P. salmonis* to cellular iron with different efficiencies between LS and HS families. In view of this, our results support that the iron-deprivation mechanism of nutritional immunity [53] could be an important defence mechanism against *P. salmonis* infection.

### Genome sequence of *P. salmonis* reveals the presence of iron-acquisition genes

To cope with iron-deprivation mechanisms, pathogens have evolved mechanisms for iron acquisition that are tightly controlled by the availability of iron in the environment [46]. Herein, we sought to identify *P. salmonis* iron acquisition genes that could act as potential virulence factors. To do this, we sequenced and annotated the genome of *P. salmonis* and identified a set of orthologous genes with reported roles in the synthesis and uptake of siderophores and heme, ferric iron active transport (energy system), ferrous iron acquisition, and transcriptional regulation (Fur, ferric uptake repressor). Some features of these genes are shown in Table 5.

**Table 5:** Iron acquisition systems of *P. salmonis*

^a^Genbank ID	^b^ORF	^c^Length (bp)	^d^Closest homolog in Genbank (blastx)	^e^E value	^f^Protein identity (%)
KJ804204	fur	444	WP_018274636.1 Fur family transcriptional regulator [*Teredinibacter turnerae*]	3.0E-70	68
KJ804205	feoA	234	YP_004591266.1 ferrous iron transport, protein A [*Enterobacter aerogenes*]	2.0E-14	51
KJ804206	feoB	2085	YP_003038990.1 ferrous iron transport, protein B [*Photorhabdus asymbiotica*]	1.0E-104	53
KJ804207	feoC	285	YP_002799440.1 ferrous iron transport, protein protein C [*Azotobacter vinelandii*]	4.0E-06	33
KJ804208	fhuA/hemeR	1974	YP_001184687.1 tonB-dependent hemin/siderophore receptor [*Shewanella sp.*]	1.0E-32	24
KJ804209	tonB	789	YP_003459692.1 tonB family protein [*Thioalkalivibrio sp.*]	4.0E-16	41
KJ804210	exbD	630	WP_021695817.1 biopolymer transport protein ExbD/TolR [*Loktanella cinnabarina*]	7.0E-06	64
KJ804211	exbB	660	YP_006295784.1biopolymer transport protein ExbB [*Methylophaga nitratireducenticrescens*]	6.0E-21	58
KJ804212	fhuD	981	NP_484432.1 periplasmic iron (III) -binding protein, ABC transporter [*Nostoc sp.*]	1.0E-18	52
KJ804213	fhuB	2037	YP_323336.1 iron-hydroxamate transporter permease [*Anabaena variabilis*]	3.0E-46	44
KJ804215	fhuC	858	WP_006640164.1 iron-hydroxamate ABC transporter ATP-binding protein [*Bacillus sonorensis*]	2.0E-61	54
KJ804218	pvuA	2190	YP_159259.1 siderophore receptor, TonB-dependent [*Aromatoleum aromaticum*]	2.0E-148	45
KJ804219	pvsA	1206	WP_018314298.1 siderophore biosynthesis [*Cupriavidus sp.*]	1.0E-79	51
KJ804220	pvsB	1335	WP_018077816.1 siderophore biosynthesis, (carboxylate-amine ligase) [*Thiobacillus denitrificans*]	4.0E-69	47
KJ804221	pvsC	1239	WP_005449425.1 Multi-drug efflux pump PvsC [*Vibrio harveyi*]	4.0E-62	35
KJ804222	pvsD	1812	YP_747943.1 siderophore biosynthesis, IucA/IucC family protein [*Nitrosomonas eutropha*]	2.0E-67	42
KJ804223	pvsE	1063	AHI32018.1 siderophore biosynthesis (diaminopimelate decarboxylase) [*Marinobacter sp.*]	4.0E-137	49

^a^GenBank accession numbers

^b^Open Reading Frame

^c^Length of gen (bp)

^d^Closest annoted homolog in Genbank by blastx against nr database

^e^ E value and

^f^Protein identity (%)

From the genome sequence, three orthologous genes were predicted encoding for synthetases of vibrioferrin, an unusual marine carboxylate siderophore (*pvsA, pvsB, pvsD*); its membrane-spanning exporter (*pvsC*); and its TonB-dependent siderophore receptor (*PvuA*) [54]. All classic components of the ExbB − ExbD − TonB energy system were found, supporting the TonB-dependent active transport of iron siderophores across the outer bacterial membrane [55]. Furthermore, components of both *fhu* and *feo* operons, required for the acquisition of hydroxamate siderophores (or heme) [56] and ferrous iron [57] respectively, were predicted from the *P. salmonis* genome. Finally, an orthologue of Fur was also identified. Fur controls the intracellular concentration of iron in bacteria, thus in the presence of intracellular iron, Fur binds DNA and represses the transcription of genes involved in siderophore biosynthesis and iron acquisition [58].

**Fig. 6:**
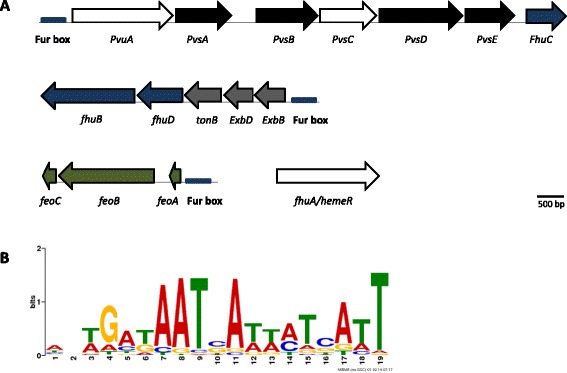
Putative siderophore biosynthesis and iron transport gene clusters of *P. salmonis* and Fur binding-site prediction. **a** Siderophore biosynthetic genes are indicated by black arrows. The congate siderophore/heme outer membrane receptor/exporter genes are depicted in white and the component of TonB-dependent active transport across the bacterial outer membrane system is in grey. Green arrows denote components involved in the ferrous uptake system and blue arrows indicate ferric hydroxamate/heme uptake genes. A single homolog of the ferric uptake regulator (fur) was predicted in *P. salmonis* genome. The blue box represents Fur-binding sites. **b** Sequence logo for predicted Fur-binding sites in *P. salmonis*

A schematic representation of *P. salmonis* iron acquisition genes in a genetic context (Fig. 6a) shows that except for *fur* and *fhuA*/*hemeR*, genes were distributed into three separate iron gene clusters. The presence of these components suggests that *P. salmonis* can acquire iron through multiples mechanisms, including those for the transport of ferric and ferrous iron, heme iron, and both endogenous and exogenous siderophores. This allows us to presume that iron is a crucial element for the survival and virulence of *P. salmonis.* The capacity of pathogenic bacteria to acquire iron in an animal host is important for establishing infection [59]. Since animal hosts have essentially no free iron but do have different heme sources, it is probable that mechanisms for heme capture are relevant during infection. Moreover, orthologous genes that encode for hemolysin and its related secretion components (*hlyb1* and *hlyb2*; *hlyd*, *tolc1* and *tolc2*) were also identified [60]. Hemolysins are cytolytic toxins to erythrocytes and other cell types that are produced by some heme-acquiring bacteria and are considered to be virulence factors [61]. The presence of these genes in the *P. salmonis* genome is consistent with the haemorrhagic and anaemic response previously described in fish during *P. salmonis* infection [5, 10].

To explore whether the predicted iron gene clusters were regulated by Fur, we sought putative Fur-binding sites in the whole genome of *P. salmonis*. For this purpose, the Fur-binding site motif of γ-proteobacteria was reconstructed from 656 manually curated intergenic sequences, and this information was used to search for this motif in the entire *P. salmonis* genome. The sequence logo for the predicted Fur-binding sites in *P. salmonis* is shown in Fig. 6b.

The results indicated that two hundred open-reading frames (ORFs) had at least one putative Fur-binding site in the intergenic region upstream of the respective start codon (data not shown). More importantly, a putative Fur-binding site was found upstream of the three iron gene clusters described above (Fig. 6a), suggesting that these gene clusters might be regulated by Fur and become activated during iron deficiency.

**Fig. 7:**
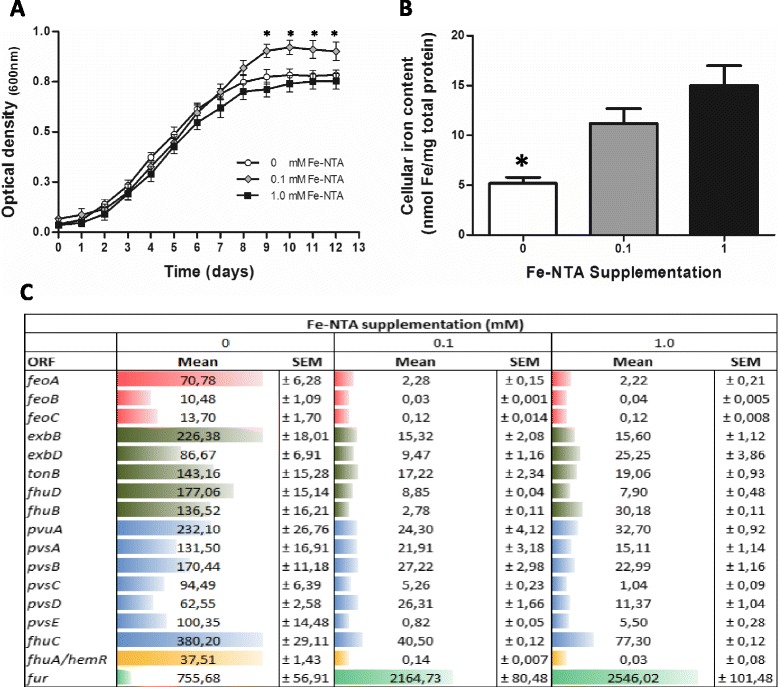
Effect of iron availability on *P. salmonis* growth and expression of iron-acquisition genes. **a** Growth curves of *P. salmonis* treated with three concentrations of iron supplementation (0 mM, 0.1 mM and 1 mM of Fe-NTA) during twelve days. Each point represents the mean of nine determinations (± SEM); *, *p* < 0.05 (Student’s *t* test). **b** Iron content of *P. salmonis* treated with three concentrations of iron supplementation (0 mM, 0.1 mM and 1 mM of Fe-NTA) during five days. Columns represent the mean of five determinations (± SEM); *, *p* < 0.05 (Student’s *t* test). Iron content was normalized towards mg of total proteins. **c** Relative expression of iron acquisition genes was determined using qPCR. These genes were significantly modulated (Student’s *t* test *p* < 0.05) in response to iron deficit in comparison with the iron supplemented conditions. The relative abundance of each mRNA was normalized towards the recombinase A (Rec A) mRNA of *P. salmonis*. Bars and numbers represent the mean value of five determinations (± SEM). Different colors represent different iron cluster genes

To address this, the bacterium was cultured for twelve days in a free-blood standard liquid media supplemented by 0.1 mM of ferric iron (Fe-NTA) (reference condition), which permitted optimum bacteria growth [62]. Two other experimental conditions were also used; one without ferric iron (0 mM) and another with 1.0 mM of ferric iron supplementation, representing deficit and excess iron availability, respectively. Data showed that carrying capacity (K) was significantly lower in both experimental conditions than in the reference condition, however no significant differences in growth rates (exponential phase, at 5 d) were observed under the different supplementation conditions (Fig. 7a). On the other hand, the intracellular bacterial contents of iron, measured by Atomic Absorption Spectroscopy (AAS) during the exponential growth phase, showed that in the absence of iron supplementation there was a significant decrease in the intracellular concentration of iron when compared to the reference condition. However, no significant differences in iron contents were detected among bacteria grown in iron supplemented conditions (Fig. 7b). These results indicate that *P. salmonis* senses iron fluctuations in the environment and adaptively responds to both iron deficiency or excess, and since both conditions are deleterious for the bacteria, expression of acquisition and detoxification systems are finely regulated [63, 64].

The expression of predicted iron acquisition genes under supplemented and non-supplemented iron conditions was also measured. A significant increase in the relative abundance of all predicted transcripts occurred in response to iron deficiency (0 mM Fe-NTA) as compared to both supplemented conditions (Fig. 7c). This behaviour was supported through a putative Fur-binding site found present upstream of the three iron gene clusters described.

Although *fhuA/hemeR* was not predicted as part of an iron gene cluster, and a Fur box was not identified in its upstream region as has been described in other bacterial species [56, 65], the transcriptional behaviour of *fhuA/hemeR* indicates that it might be part of a still un-annotated polycistronic operon regulated by Fur. However, future assays are necessary to confirm this hypothesis.

Interestingly, the abundance of transcripts that encoded a putative Fur transcription factor was significantly reduced in the iron-restricted condition. The absence of a predicted Fur-binding site, and the role of Furas a repressor of iron acquisition genes, support its transcriptional behaviour, which has also been reported in other bacterial species [66, 67]. Finally, from previously published information and the present results, we formulated a model for the possible spatial organization of the predicted proteins involved in iron acquisition in *P. salmonis* (Additional file 7). In light of the severity of *P. salmonis* infection and the risk of acquiring antibiotic-resistant bacterial strains, fully describing iron acquisition systems and the roles of these in *P. salmonis* pathogenesis is a crucial step towards developing therapeutic agents.

To the best of our knowledge, this is the first study that compares the transcriptional response to *P. salmonis* infection among Atlantic salmon families with different levels of susceptibility. Nevertheless, this study has some limitations, for instance, fish were infected by intra-peritoneal injection, a method that does not represent the natural form of *P. salmonis* infection. Moreover, for gene expression analysis, a single tissue (head kidney) was sampled and only at one time-point, so we could have missed other differentially expressed transcripts that appear in other tissues affected by the infection and before and after 14 dpi. Such issues can be addressed by using different infection protocols, multiple tissues and time-point measurements, and in future studies, by using RNA Sequencing in order to identify potential splicing variants and polymorphisms among salmon families, information that could be relevant to understand fish resistance to infection. Another limitation of our study is that the complexity of *P. salmonis* genome only permitted us the assembly of a draft genome (198 scaffolds). Thus, in order to identify the complete set of genes involved in iron metabolism and acquisition and their genetic contexts, further studies are necessary to assembly the complete *P. salmonis* genome.

## Conclusions

We carried out experimental infection challenges against *P. salmonis* in Atlantic salmon families*.* Results revealed a heterogeneous distribution of cumulative mortality among families that may be explained by the genotype of the challenged families. To further explore these differences, we compared the transcriptional response between groups of families with different susceptibilities to *P. salmonis* infection. Our results revealed distinctive gene expression profiles between LS and HS groups of families, and this led to the identification of biological processes possibly involved in natural resistance. In particular, the ability of fish from LS families to limit iron availability to the bacterium suggests that iron-deprivation represents a mechanism of innate immunity and resistance against *P. salmonis*. These data were complemented with predictions of iron acquisition systems in the *P. salmonis* genome. Identification of putative Fur boxes and the expression of these genes under iron-depleted conditions revealed that most form part of the Fur regulon of *P. salmonis.* This information contributes to current understands on the varied responses of fish with different levels of susceptibility to *P. salmonis* infection and supports the development of future treatment strategies.

## Methods

### Fish and infection experiments

Forty unvaccinated F1 full-sibling families of Atlantic salmon (mean weight 276.9 ± 78.3 g and length 283.6 ± 25.4 at start), were obtained from Agua Buena Center, Puerto Montt, Chile. Fish were implanted with a Passive Integrated Transponder (PIT) tag containing a specific code to identify each individual fish and thus the family to which they belong. Fish were maintained in 7 m^3^ tanks at enterprise SGS Chile Ltda., Puerto Montt, Chile, with seawater in a recirculation system at 14.7 ± 0.1 °C water temperature, 33.92 ± 0.04 ppm salinity, and 93.35 ± 1.28 % oxygen saturation, sharing the same nutritional (frequency, amount and type of food) and environmental (same tanks) conditions. Fish were randomly tested and screened to ensure that they were pathogen-free (SRS, IPNv, BKD and ISAv).

Two challenges with *P. salmonis* were performed. For the first challenge up to around twenty fish from each family were randomly transferred to ten tanks of 0.75 m^3^ and kept for four weeks in acclimatization under the conditions listed above. Then, all fish were anesthetized with benzocaine (Sigma) (20 % w/v; 50 mg/L) and immediately infected by intra-peritoneal injection with 0.2 ml of *P. salmonis* (PS889, isolated from *Oncorhynchus kisutch* on 2003) (1 × 10^4^ PFU/ml) in minimal essential media (MEM). *P. salmonis* inoculum was grown in CHSE-214 cell cultures (ATCC catalog number: CRL-1681) with 80-90 % cell confluence, according to standard operating procedures of SGS Chile Ltda. During the challenge, experimental fish were observed daily and mortality per family group was recorded daily for 40 days post-infection. After the forty days, fish were harvested and the percentage of cumulative mortality (dead fish/total fish*100) for each family was calculated. For the second challenge, families with the highest cumulative mortality (greater than 30 %) levels were considered of relatively high susceptibility (HS) and families with the lowest cumulative mortality (0 %) levels were considered of relatively low susceptibility (LS) to *P. salmonis* infection. For the second challenge, representative naive fish from six LS and six HS families were selected. Then, ten fish from each family were anesthetized and inoculated by intra-peritoneal (IP) injection of *P. salmonis* (experimental condition, infected fish), using the conditions previously described. The remaining ten fish were injected with sterile culture media, representing the control condition (non-infected fish). Fish were homogeneously distributed in three tanks of 0.7 m^3^, and acclimatized as described above. Fourteen days post-injection (dpi) all fish were sacrificed by overdose of benzocaine (100 mg/L during 10 min) and their head kidneys were removed using aseptic techniques, stabilised in RNAlater (Ambion) at 4 °C for 24 h and then stored at −80 °C until RNA extraction. To confirm the presence of *P. salmonis* in Atlantic salmon tissues, DNA was extracted from head kidney of all fish by DNEasy kit (Qiagen) and used as template in a qPCR reaction as described by Karatas et al. [68]. The trials were approved by the Ethics Committee of the Institute of Nutrition and Food Technology, University of Chile.

### RNA isolation

For microarrays hybridizations, total RNA was extracted from 50 to 100 mg of head kidney using TRIZOL Reagent (Invitrogen), and incubated for 30 min at 37 °C with 20 units of RQ1 RNase-Free DNase (Promega) to remove residual genomic DNA. Then, RNA was purified using RNeasy mini kit (Qiagen) according to the manufacturer’s instructions. Even though we selected six LS and six HS families for the second challenge, we were able to obtain five individual samples of good RNA quality only from fish of three LS and three HS families of those six families.

For qPCR of iron acquisition genes of *P. salmonis*, total RNA was extracted with the RiboPure Bacteria Kit (Ambion). Pellets form liquid cultures of bacterium were re-suspended in 350 μl of RNAwiz solution and transferred to a 0.5 ml RNAse-free skirted screw-cap microcentrifuge tube containing 250 μl of 0.1 mm ice-cold Zirconia Beads. Then, tubes were placed horizontally on the vortex adapter and processed at maximum speed during 15 min. Remaining steps were performed according to the manufacturer’s instructions.

All RNA samples were treated with Dnase I (Ambion) and purified by RNeasy column (Qiagen) The quantity of the total RNA were determined using a Qubit Fluorometric Quantitation System (Life Technologies) and the purity (absorbance 260/280 nm) using a NanoQuant Spectrophotometer (Tecan Technologies), while integrity was confirmed by RNA Integrity Number (RIN) using a 2200 TapeStation Instrument (Agilent Technologies, Inc). Only samples of high quality (Absorbance 260/280 nm ≥ 1.8 and RIN ≥ 8.0) were used for gene expression analyses. After quality analysis of the RNAs, the best individual samples from both infected and non-infected fish of each family were selected.

### cDNA synthesis and labelling

For microarrays hybridizations, pools of RNA from five control and five infected fish of each one of the six families were prepared separately by mixing the same mass of RNA from each individual sample. Three μg of the pooled RNA were reverse transcribed using a SuperScript® Indirect cDNA Labelling System (Invitrogen). Briefly, cDNA synthesis was performed at 46 °C for 3 h in a 12 μl reaction volume, followed by RNA degradation with 1 M NaOH at 37 °C for 15 min and neutralization with 1 M HCl. Then, cDNA was precipitated with ethanol and sodium acetate (3 M, pH 5.2) overnight at −20 °C and the pellets obtained were washed in 70 % ethanol and air dried before resuspension in 5 μl 2X coupling buffer. Once the cDNA was fully dissolved, 2 μl of Cyanine 3 (Cy3-dUTP) or Cyanine 5 (Cy5-dUTP) (GE HealthCare; PA23001, PA25001) dyes was added and incubated in the dark overnight at 25 °C. To remove unincorporated dye, labeled cDNA was extracted with phenol:chloroform:isoamyl alcohol (25:24:1) and then precipitated with ethanol and sodium acetate (3 M, pH 5.2) overnight at −20 °C. The pellets were washed with 70 % ethanol and air dried before resuspension in 25 μl of hybridization buffer (50 % Formamide, 8X SSC, 0.5 % SDS, 4X Denhardt). Dye incorporation was checked by spectrophotometry.

### Microarray hybridization

Labeled samples were hybridized to a 32 K cDNA microarray developed at the Consortium for Genomics Research on All Salmonids Project (cGRASP), University of Victoria [69] (GEO accession number: GPL8904). The cGRASP microarray contains 27,917 Atlantic salmon and 4065 rainbow trout cDNA features from different cDNA libraries. Before hybridization, slides were pre-treated with 3 % BSA fraction V, 5 X SSC, 0.1 % SDS for 90 min at 49 °C and washed with 5X ultrapure water for 30 s at room temperature. cDNA samples from control and infected fish of each family were mixed, heated at 90 °C for 2 min and hybridized to the microarrays for 16 h at 49 °C in a water bath. Four slides were used for each family cDNA and hybridizations used a dye-swap design. Next, hybridization slides were washed five times with washing solution A (2X SSC, 0.2 % SDS) for 10 min at 49 °C, twice with washing solution B (1 X SSC) for 10 min and four times with washing solution C (0.1 X SSC) at room temperature and immediately dried by centrifugation before scanning.

### Statistical analysis of microarrays

Microarrays slides were scanned using a Scan array Express system (PerkinElmer), all slides were scanned with set laser power at 90 % and photomultiplier gain at 55 % for Cy5 and Cy3. A group of measures including foreground (FG) and background (BG) values of intensity for each spot in both channels were stored as tab-separated text file for further analysis. Quality of microarray images was examined by inspection of FG and BG distribution of red (R) and green (G) channels in each slide. Spearman correlation between R and G channels from replicate microarrays was calculated in order to select unbiased microarray data for significance analysis. For selected microarrays, a quality index based on local and global BG values (adapted from [70]) was used to filter and eliminate spots with low quality.

Microarray analyses were conducted separately for fish with high or low susceptibility to infection using each of the three LS families and each of the three HS families as biological replicates. Therefore, for each group of families, LS and HS, we carried out 12 microarray hybridizations, which can be divided into three independent biological replicates (the three different families of each group), each with 4 pseudo-replicates. Prior to significance analysis of data, we transformed the foreground values of red and green channels into a matrix of log intensity ratios (M) and the geometric average between channels intensities (A). Data were background corrected using a *normexp* procedure which fits a convolution model of normal and exponential distributions to the foreground intensities using the background intensities as a covariate [71]. After this, data were normalized using an intensity dependent normalization procedure (loess) [72]. Independent linear models for LS and HS families were fitted to log_2_ expression data for each gene across the microarrays [73]. In each case, the coefficient of the model represented the estimated fold change between infected and non-infected samples. These values were used to compute a moderated t-statistics, which has the same interpretation of an ordinary t-statistic except for the standard errors that have been moderated across genes using a simple Bayesian model. Raw p-values were corrected for multiple testing using a false discovery rate (FDR) of 0.05. Genes with an adjusted p-value under this cut-off were acknowledged as differentially expressed. All calculations were performed on R statistical software environment running on Linux or Windows machine, using *limma* package [74]. Microarray data were submitted to Gene Expression Omnibus (GEO, accession number: GSE43255).

### Real time PCR (qPCR)

Reactions were carried out on a Light Cycler System 1.5 (Roche Diagnostics) using LightCycler FastStart DNA Master SYBR Green kit (Roche Applied Science). Pooled RNA of each fish family, previously used for microarray analysis, was used to verify a set of differentially expressed genes from the microarray experiment and to evaluate relative abundance of iron genes between infected and control fish. To accomplish this, 2 μg of total RNA was used as template for reverse transcription reactions to synthesize single strand cDNA using MMLV-RT reverse transcriptase (Promega) and oligo-dT primer (Invitrogen), according to standard procedures. Similarly, cDNA of *P. salmonis* was synthesized from 2 μg of total RNA incubated by 1 h at 37 °C using High Capacity RNA to cDNA kit (Applied Biosystems), following manufacturer instructions. cDNAs were diluted to 100 ng and used as template for qPCR, with primers designed against genes of interest. PCR conditions were 95 °C for 5 min followed by 94 °C for 15 s, 57–60 °C for 15 s and 72 °C for 20 s for a total of 35 cycles. Melting curves (1 °C steps between 75–95 °C) ensured that a single product was amplified in each reaction. To determine relative expression levels of genes, the method described by Pfaffl [75], and adapted by Talke et al. [76] was used. Elongation factor 1 alpha (EF1A) of Atlantic salmon and recombination protein F (*RecF*) of *P. salmonis* were selected as internal reference genes. At least five replicates were performed and differences among conditions were analysed using Student’s *t*-test (*p* < 0.05) or One-way ANOVA. PCR efficiencies were determined by linear regression analysis performed directly on the sample data using LinRegPCR [77]. Additional file 8 shows the complete list of primers used in this study.

To validate the results from the microarray experiments, 40 genes were randomly selected from the microarray data and their expression levels were determined by qPCR. For each gene, mean log_2_ ratios (infected/control) determined by microarrays were plotted against mean log_2_ ratios (infected/control) determined by qPCR assays. Correlation between microarrays and qPCR were calculated by Pearson product moment correlation and a *p* < 0.01 was considered statistically significant.

### Functional analysis and clustering

To carry out the functional analysis, we uploaded the significant differentially expressed probes into Cytoscape (v3.1.1) [78]. Using ClueGO (v2.1.3) plugin, [79] we performed an enrichment analysis based on functional classifications available at Kyoto Encyclopedia of Genes and Genomes (KEGG) [80], Reactome [81] and Gene Ontology (GO) [82], using the complete set of probes of 32 K cDNA microarray as reference. Probes were previously re-annotated by *Danio rerio* RefSeq Database (ensembl 9.6 release). Enrichment was tested based on a hypergeometric test with p-value corrected by Benjamini-Hochberg method, and functions with adjusted *p* < 0.05 were selected.

Hierarchical Clustering Analysis was performed using Multi Experiment Viewer software (MeV v4.4.0) [83, 84]. From the total list of differentially expressed probes in the two groups of families, LS and HS, a gene expression matrix was built using the fold change values of each gene in each one of the six families. When a differentially expressed probe in a family had a missing fold change value (NA) in another family of its group (LS or HS), we used the average of the two remaining families of the group. If a differentially expressed probe had a missing fold change value in two families of its group, the probe was not incorporated into the analysis. Pearson correlation (Additional file 5: Figure S1A) and Euclidian distance (Additional file 5: Figure S1B) were used as distance metrics and an average linkage clustering as a linkage method.

### Cellular iron content

For total iron (Fe) content quantification, 50 to 100 mg of five head kidney tissues, representatives of control and infected conditions from LS and HS families, were dried by evaporation (SpeedVac, Thermos) and processed as described in [85]. Briefly, tissues were mechanically disrupted using a homogenizer (Omni International 2000) in concentrated nitric acid Suprapur (Merck, Chemical Co.) and digested at 80 °C overnight. Then, samples were diluted with ultrapure distilled-deionised water (Merck, Chemical Co.) until a final nitric acid concentration of 5 % v/v.

For determining iron content of *P. salmonis,* bacteria were processed as described in [86]. Briefly, bacteria were collected by centrifugation at 7800 g for 15 min, and washed once with phosphate-buffered saline (pH 7.0) and twice with ultrapure distilled deionised water. Washed cells were mixed with 1 ml of concentrated nitric acid Suprapur and 0.1 ml of concentrated perchloric acid (Ultrapure AA-100; Tama Chemicals). Cells were dissolved into liquid by microwave treatment. Then, samples were dissolved in 1 ml of 5 % v/v nitric acid solution.

Iron content was determined by means of a graphite furnace atomic absorption spectrophotometer (Perkin Elmer, SIMMA 6100, Shelton, CT, USA). Calibration was performed against an iron standard curve and iron content values (μg) were expressed per mg of dry weight tissue (kidney) or mg of total proteins (*P. salmonis*). Zinc (Zn) content measurement was performed under the same conditions. The difference between conditions was analysed using Student’s *t*-test (*p* < 0.05).

### Bacterial growth

*P. salmonis* was grown aerobically in A broth (AB) medium [62] supplemented with 0.1 % L-Cys (Sigma), 2.5 % FBS (Gibco), 1.5 % NaCl (Merck) and (0 mM, 0.1 mM or 1 mM of Fe-NTA) (Sigma) at 17 °C with shaking (100 rpm). Growth was measured by monitoring the optical density at 600 nm (OD_600_) every day during 12 days to build the growth curve. Each point in the curve represents the mean of nine determinations (± SEM), differences among conditions were analysed using Student’s *t*-test (*p* < 0.05).

### Genome sequencing of *P. salmonis*

For genome sequencing, the chromosomal DNA of *P. salmonis* cultured in liquid medium, was extracted using the DNeasy blood and tissue kit (Qiagen). The draft genome sequence of *P. salmonis* LF-89 strain was determined using a 5 Kb mate pair library with the Roche 454-GS FLX Titanium and a shotgun library with a Roche 454-GS Junior GS, reaching a 17-fold coverage of the genome. Using wgs-assembler (Celera v.7) [87], with default parameters, were produced a total of 198 scaffolds (N50 scaffold size was ∼ 38 Kb), with an average length of 15 Kb. The longest and shortest scaffolds were 140 Kb and 1 Kb, respectively. Potential Coding DNA Sequences (CDS) were identified with REGANOR [88], and the genome sequence was annotated in a locally installed annotation system GenDB v2.2 [89]. The draft genome was 2,988,956 bp in length and contained 3173 CDS. The Whole Genome Shotgun project has been deposited at DDBJ/EMBL/GenBank under the accession AMGC00000000. The version described in this paper is the first version, AMGC01000000.

### Bioinformatic prediction of Fur box

Manually curated (*n* = 656) intergenic sequences of Fur regulons from γ-proteobacteria stored in RegPrecise 3.0 database [90] were used to reconstruct the Fur motif using the MEME software [91] (parameters –dna –w 19 –minsites 650). Then we searched this motif against intergenic regions of the annotated genome of *P. salmonis*, using the MAST software [92]. The results from this analysis were manually curated.

### Availability of supporting data

The Whole Genome Shotgun project has been deposited at DDBJ/EMBL/GenBank under the accession AMGC01000000 (http://www.ncbi.nlm.nih.gov/bioproject/199690)

Microarray gene expression data is available at GEO at the NCBI (GenBank) under the accession GSE43255 (http://www.ncbi.nlm.nih.gov/geo/query/acc.cgi?acc=GSE43255)

All nucleotide sequences of iron acquisition genes are available at NCBI GenBank under accession numbers KJ804204 to KJ804223 (http://www.ncbi.nlm.nih.gov/nuccore).

All other data sets supporting the results of this article are included within the article and its additional files.

## Additional files

Additional file 1:
**Schematic diagram of experimental design.**
Additional file 2:
**Complete list of differentially expressed probes between infected and non-infected fish from LS and HS families.** Table displays the ^1^GenBank accession numbers, ^2^tentative annotation of each probe differentially expressed (*p* < 0.05) between infected and non-infected fish of both LS and HS families and their ^3^fold change (log_2_) expression ratios obtained from microarray analysis. Additional file 3:
**Complete list of genes validated by qPCR in the six families.** Table displays the ^1^LS or HS family, ^2^GenBank accession numbers, ^3^Description of the best match according to BlastX or BlastN against nr GenBank database. ^4^ Fold change expression ratios between infected and non-infected fish as measured by qPCR. ^5^Fold change expression ratios between infected and non-infected fish obtained from microarray analysis. Additional file 4:
**Complete list of common probes differentially expressed in both LS and HS families following** ***P. salmonis*** **infection.** Table displays the ^1^GenBank accession numbers, ^2^tentative annotation of each up- and down-regulated probe (*p* < 0.05) between infected and non-infected fish. ^3^Fold change (log_2_) expression ratios in LS families and ^4^fold change (log_2_) expression ratios in HS families obtained from microarray analysis. Additional file 5:
**Hierarchical clustering of data from the microarray analysis.** Complete lists of differentially expressed probes were used for clustering analysis (rows). Each column represents the pattern of transcriptional response to infection of a single family. A correlation analysis was applied to measure the degree of association among the gene expression patterns of the six families using a Pearson correlation (Figure S1A) and Euclidian distance (Figure S1B) as distance metrics and an average linkage clustering as a linkage method. The color code represents the log_2_ of the expression ratio, where red represents up-regulation; green represents down-regulation, and black represents no change. Additional file 6:
**Complete list of probes with contrasting expression patterns between LS and HS groups of families.** Table displays the ^1^GenBank accession numbers, ^2^tentative annotation of probes that were significantly up- (Up LS and NM HS) or down- (Down LS and NM HS) regulated, in the LS but not in HS group of families and probes that were up-regulated in LS and, simultaneously, down-regulated in HS families (Up LS and Down HS). Additional file 7:
**Model of the iron-acquisition systems of** ***P. salmonis*** **.** The model represents the spatial distribution of proteins associated with iron acquisition in *P. salmonis*. The proposed location for each protein was estimated according to the location of orthologous proteins in other bacteria. Additional file 8:
**Complete list of primers used in this study.**
