# Supplementary figures and images for: Transcriptional response of Atlantic salmon families to Piscirickettsia salmonis infection highlights the relevance of the iron-deprivation defence system
Source: BMC Genomics. 2015 Jul 4;16(1):495. doi: 10.1186/s12864-015-1716-9 (PMC4490697; doi:10.1186/s12864-015-1716-9)

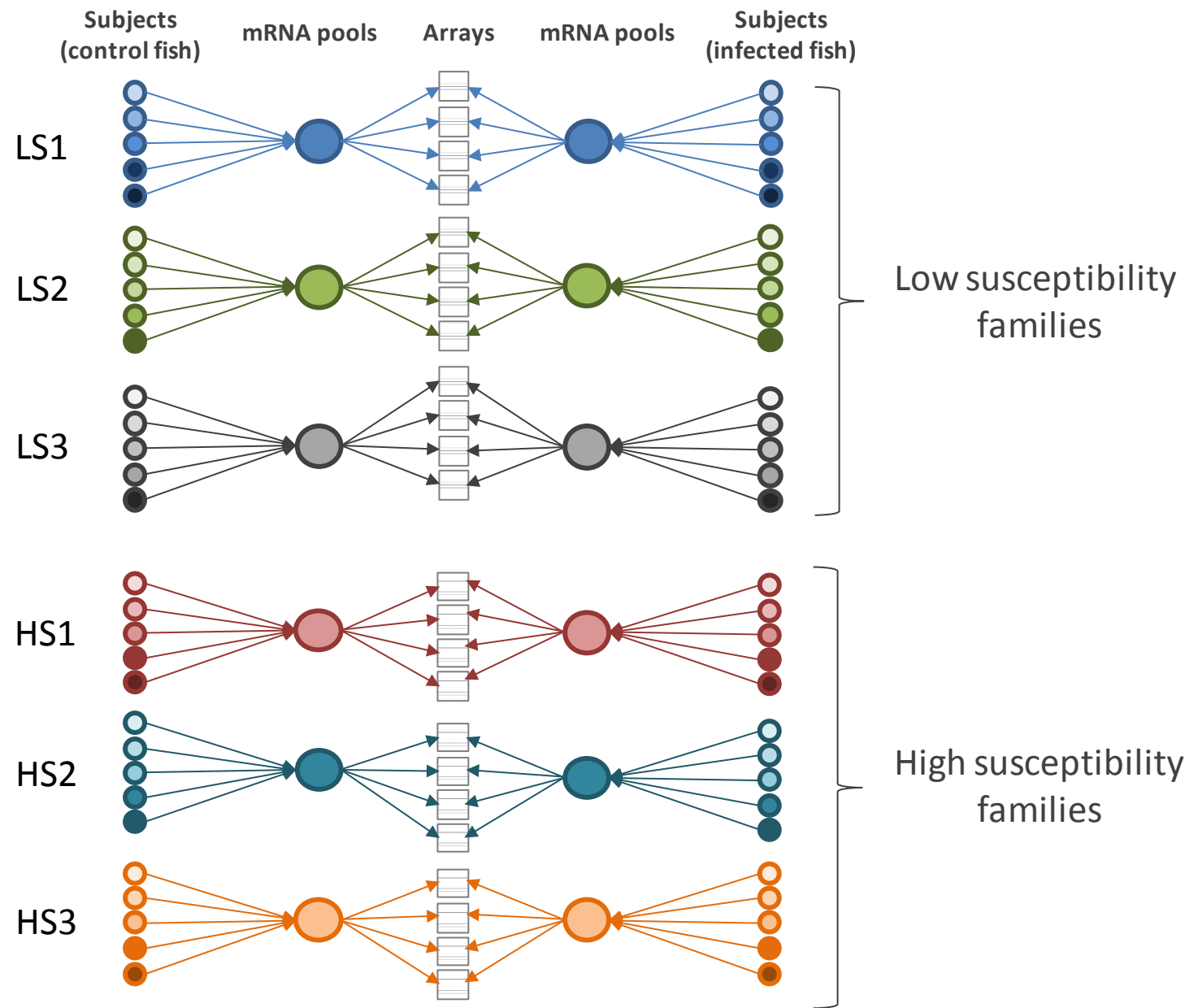

Supplement: Additional file 1: — Schematic diagram of experimental design. [file 12864_2015_1716_MOESM1_ESM.pdf]

**A**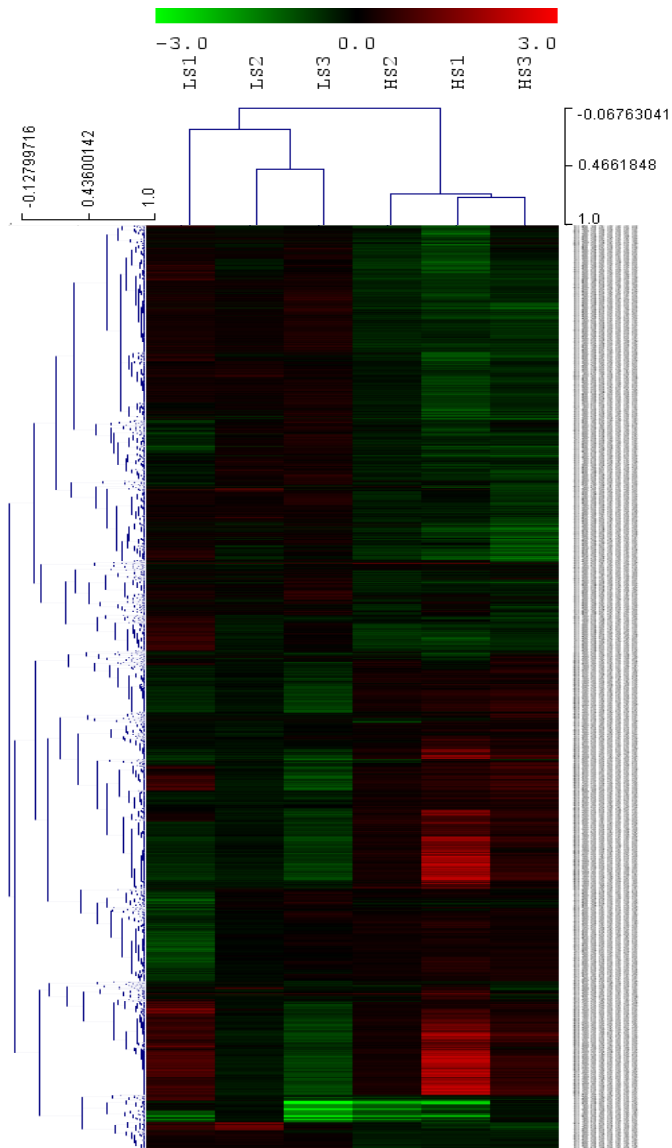**B**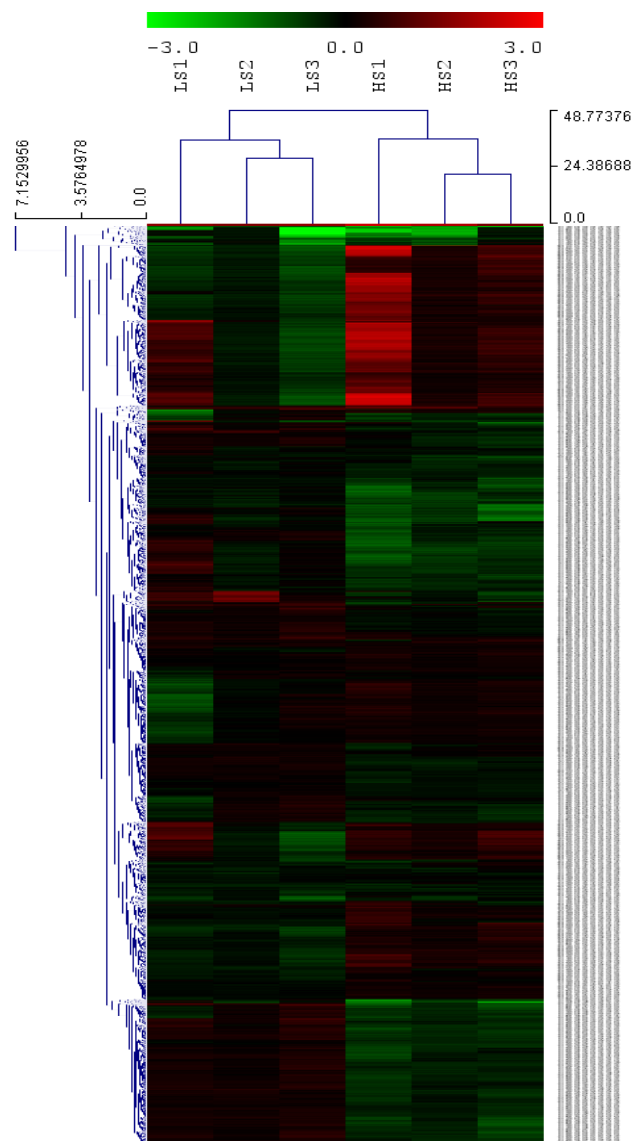

Supplement: Additional file 5: — Hierarchical clustering of data from the microarray analysis. Complete lists of differentially expressed probes were used for clustering analysis (rows). Each column represents the pattern of transcriptional response to infection of a single family. A correlation analysis was applied to measure the degree of association among the gene expression patterns of the six families using a Pearson correlation (Figure S1A) and Euclidian distance (Figure S1B) as distance metrics and an average linkage clustering as a linkage method. The color code represents the log2 of the expression ratio, where red represents up-regulation; green represents down-regulation, and black represents no change. [file 12864_2015_1716_MOESM5_ESM.pdf]

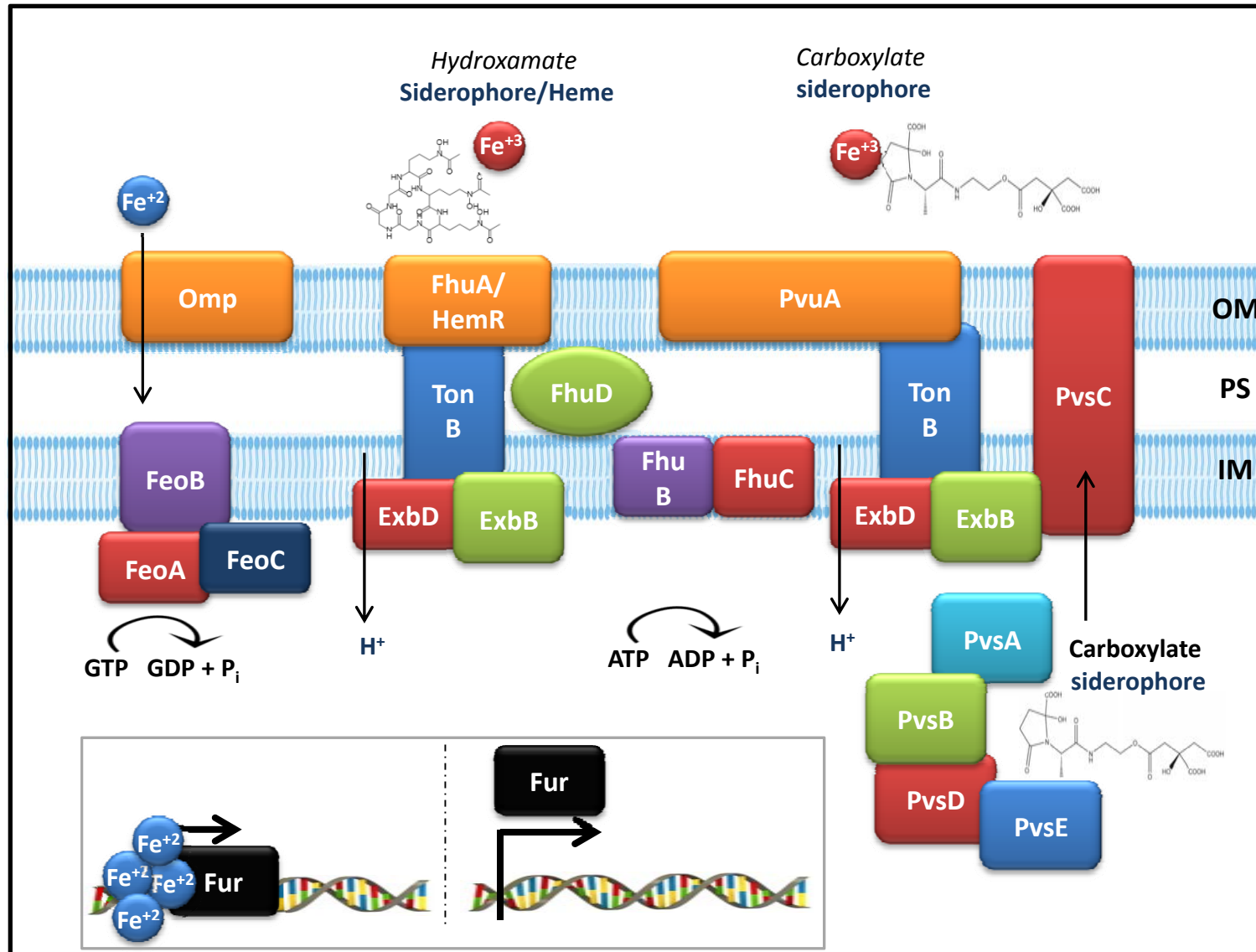

Supplement: Additional file 7: — Model of the iron-acquisition systems of P. salmonis . The model represents the spatial distribution of proteins associated with iron acquisition in P. salmonis. The proposed location for each protein was estimated according to the location of orthologous proteins in other bacteria. [file 12864_2015_1716_MOESM7_ESM.pdf]
